# Autism Case Report: Cause and Treatment of “High Opioid Tone” Autism

**DOI:** 10.3389/fpsyg.2021.657952

**Published:** 2021-05-24

**Authors:** Vishal Anugu, John Ringhisen, Brian Johnson

**Affiliations:** SUNY Upstate Medical University, Syracuse, New York, NY, United States

**Keywords:** autism, neurobiological systems engineering, case report, opioid tone, cold pressor test

## Abstract

**Introduction:** Neurobiological systems engineering models are useful for treating patients. We show a model of “high opioid tone” autism and present a hypothesis about how autism is caused by administration of opioids during childbirth.

**Main Symptoms:** Clinical diagnosis of autism in a 25 year old man was confirmed by a Social Responsiveness Scale (SRS) self-rating of 79, severe, and a Social Communications Questionnaire (SCQ-2) by the patient's father scoring 27. Cold pressor time (CPT) was 190 seconds—unusually long, consonant with the high pain tolerance of autism.

**Therapeutic Intervention and Outcomes:** At naltrexone 50 mg/day SRS fell to 54 and SCQ-−2–9; both non-significant. CPT fell to 28, repeat 39 s. Improved relatedness was experienced ambivalently, understood as feelings never before experienced—causing pain. Non-compliance with naltrexone was followed by cutting open his palm and drinking alcoholically. Transference focused psychotherapy has helped him remain naltrexone—compliant while he works on issues of identity and relatedness.

**Conclusion:** The model suggests studies that could be conducted to both prevent and treat this form of autism.

## Introduction

Autism Spectrum Disorder (ASD) is a neurodevelopmental disorder characterized by poor social skills, repetitive behaviors and nonverbal communication. The cause is obscure. The genetics are complex with gene-wide association studies showing many biomarkers. (Grove et al., 2019).

A “three hit” model of autism posits:

- A genetic neurodevelopmental vulnerability. Common and rare genetic variants contribute to ASD etiology. There are differences in polygenic architecture across clinical subtypes. Genome-wide association studies show five loci on chromosomes 1, 3, 5, and 7 (Grove et al., 2019).
- An environmental stressor that interacts with the genetic vulnerability.
- With these in place, development is adversely affected (Hendren, 2020).

Panksepp et al. discovered autism features high circulating C-terminal beta endorphin. Sixty-seven children with autism had levels that were broadly distributed but averaged 10 times higher than normal controls (Leboyer et al., 1994). Treatment with naltrexone to block opioid receptors was first carried out by Panksepp et al. Results indicated a reversal in autistic behaviors and gaze aversion at doses of 0.5, 1.0, and 2.0 mg/kg. (Lensing et al., 1995) There was no consensus regarding the optimal dose. (Campbell et al., 1989) The few published reports on naltrexone for autism found benefit for some patients but not for others (Campbell et al., 1993).

Endogenous opioids circulate through the blood. Receptors exist on white blood cells, keratinocytes, synovial cells, gut, and multiple sites in the central nervous system (Johnson et al., 2014). Endogenous opioids regulate human closeness (Panksepp et al., 1997).

On our Addiction Medicine Service at Upstate Medical University we use neurobiological engineering models to guide treatment (Johnson, 2018). Psychoanalytic models are built on clinical interactions. Neuropsychoanalysis uses neuroscience as the basic science of psychoanalysis (Johnson and Flores Mosri, 2016). Neuropsychoanalytic models should be congruent with both psychoanalytic clinical observations and when available, neuroscience (Solms and Turnbull, 2002; Johnson, 2008). A “system” as defined by the International Council on Systems Engineering is, “A construct or collection of different elements that together produce results not obtainable by the elements alone” (International Council on Systems Engineering, 2006). Neurobiological systems engineering combines complex neuroscience, abstract and simplified models based on use of neuroscience, psychoanalytic concepts, and social awareness such as the immense profit obtained by selling addictive drugs legally, to build models that guide clinical interventions (Johnson, 2018).

We will give an example next of an updated model that provides an explanation for vulnerability to development of addictive illness, autistic unrelatedness during opioid use, and symptoms of the genetic variant of autism that features high circulating C-terminal beta endorphin. This model employs a quadratic equation to schematically show our understanding of pleasure-pain and human closeness regulating endogenous opioid tone. An underlying principal is calculus seems to describe the natural world (Strogatz, 2019).

Pleasure (*x*) = 4 – (*x*−3)^2^

*x* = opioid tone, limit *x* = 0 < *x* < 6

**Figure 1:**
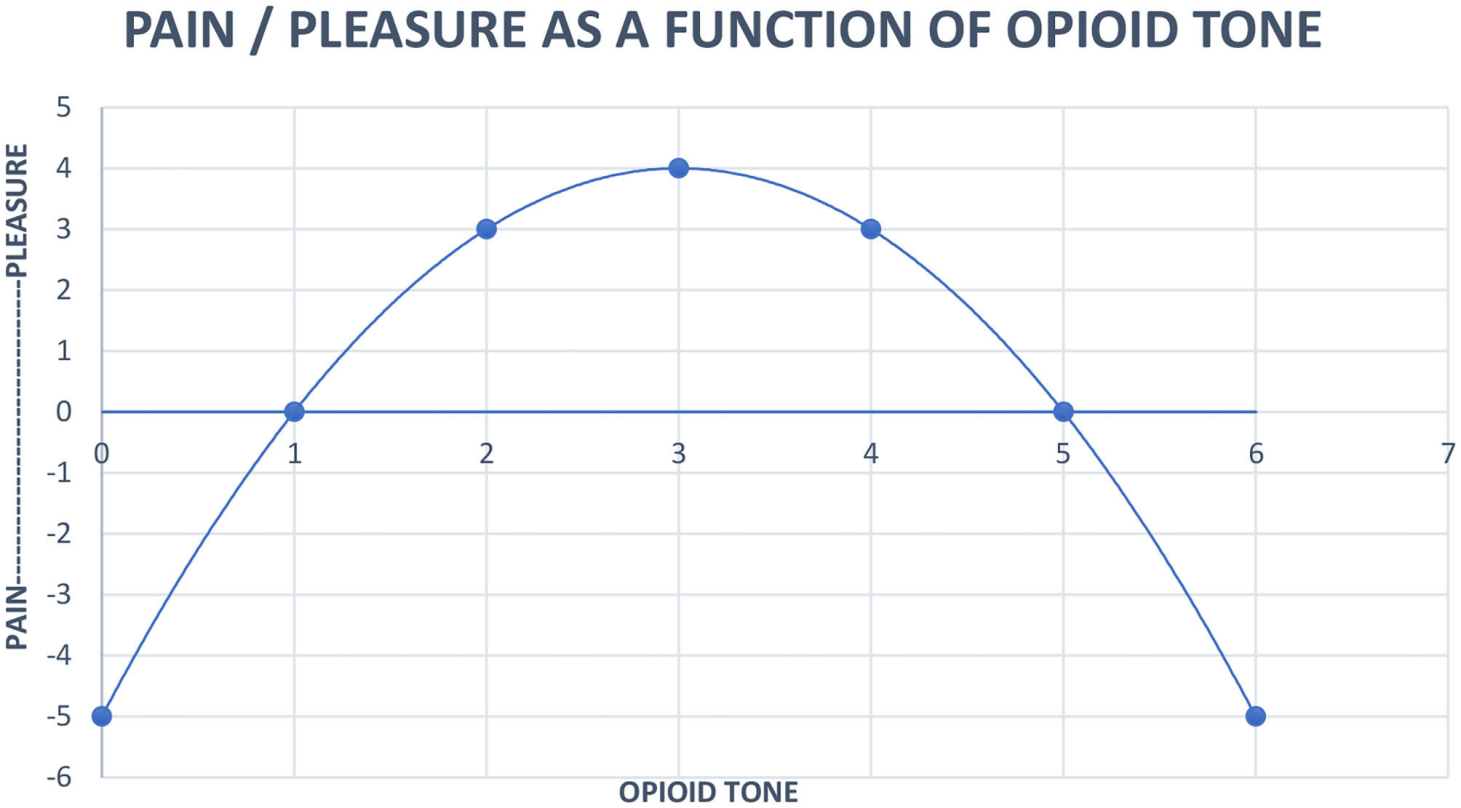
Neurobiological systems engineering model of the relationship of pain, pleasure and opioid tone.

This equation represents our clinical observations as follows:

- When healthy persons feel the distress of loneliness they seek comfort through the proximity of others to increase opioid tone. It feels good.
- Prolonged intense contact causes discomfort. Healthy humans seek solitude to reduce high opioid tone to a pleasant level.
- Human contact is used to modulate opioid tone between 2 and 4 on Figure 1. Healthy persons can engage and disengage flexibly.
- Autism's high endogenous tone, between 5 and 6 on Figure 1, causes ordinary human contact to be uncomfortable. Autistic persons reduce their opioid tone to a less painful level by seeking solitude from ordinary interactions. Healthy individuals contact with each other by looking at each other, talking to each other, and touching each other, while gaze avoidance, lack of social engagement, and repetitive behaviors meant to disengage characterize autism. Autistic patients don't want to be touched.

Increasing opioid prescribing is correlated with increasing autism. In our 2014 review of obstetric literature, moving from the “natural childbirth” ideal of the 1970s to the American College of Obstetrics and Gynecology encouraging opioid administration during childbirth in the 2000s, we suggested opioids were resetting endogenous opioid tone and causing autism in genetically vulnerable newborns. (Johnson et al., 2014) We offer this as a possible cause while appreciating that correlation does not require causation.

**Figure 2:**
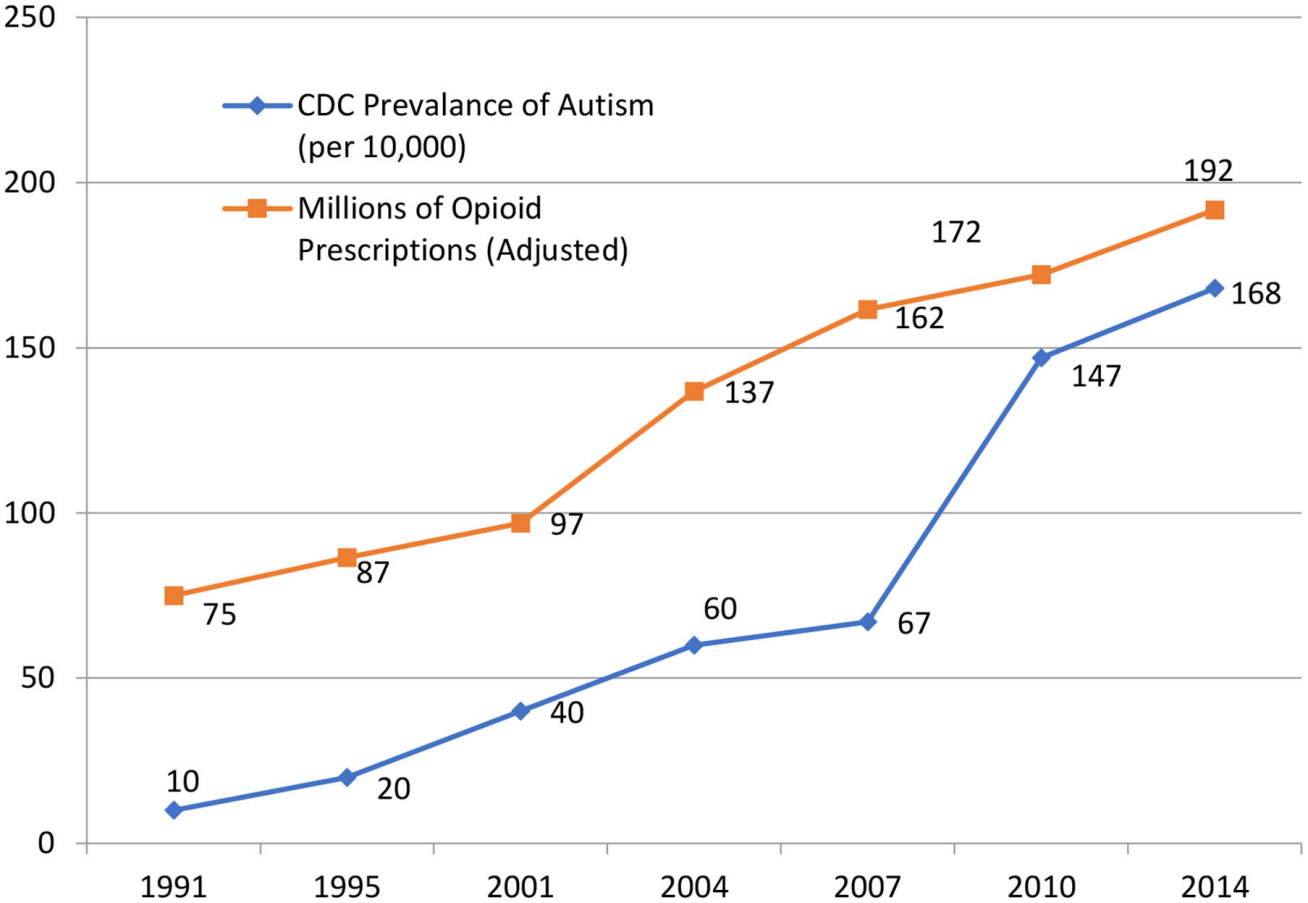
Autism rate and opioid prescription correlation. Correlation *p* < 0.001.

We have updated findings from the 2014 paper, now covering from 1991 until 2014 and still find the correlation between the Centers for Disease Control prevalence of autism and millions of opioid prescriptions in the United States *p* < 0.001 (Figure 2, Table 1).

**Table 1:** Population-adjusted rates of autism and opioid prescribing in the United States *adjustment based on 1991 population.

**Days**	**0**	**8**	**28**	**35**	**42**	**49**	**55**	**65**	**98**
Intervention	Assessment	CPT	Naltrexone	Naltrexone				Resumed naltrexone	
			25 mg	50 mg					
Result	MDD	190 s	Better	Even Better	CPT	CPT	Lost naltrexone	Again better	Hired full time
	ADHD				28 s	39 s	Cut open palm−11 sutures to close wound		
	BPD								
	Alcohol, Cannabis, Tobacco use disorder								

We show the use of the neurobiological systems engineering model of Figure 1 in a case report.

## Case Description

A, a 25-year-old man with alcohol use disorder, presented with a chief complaint of feeling “lonely and frustrated.” His father was his sober support person.

Birth was reported difficult with more than 12 h of labor and use of opioids for analgesia. Past psychiatric history was significant for ADHD, and “anxiety, and depression,” his pediatrician had diagnosed when he was 16. He described family as “disconnected” since his parents' divorce. He felt “trapped” while living with his mother. Therefore he relocated and has lived with his father, “Since some time just after [high school] graduation.” He described distant sibling relationships “because they all hate my dad for some reason. Because I'm the only one giving him a chance they don't talk to me.”

A described a long history of poor communication and difficulty socializing. Throughout his education he was in “special classes” that he feels were stigmatizing and responsible for “making it really difficult to have friends at school. Everyone looked at us like we were weird and didn't want to be seen with us.” After high school, A worked as a custodian for a few months at a college until, for unclear reasons, he was terminated and barred from the campus. A stated, “I just decided that being alone all the time, at work and at home, wasn't good for me and never went back.” This was his only significant work in the seven years since high school graduation.

He spent much of his time indulging in alcohol, tobacco, and cannabis and avoiding social interactions. Alcohol consumption had been responsible for a hospital admission for withdrawal, an inpatient rehab followed by six months in a half-way house, and a drinking dream, suggestive of alcohol changing his ventral tegmental dopaminergic SEEKING system to produce alcohol craving (Johnson, 2001, 2003).

During the initial interview he described feeling nauseous and agitated as we discussed his relationship with addicting substances. His mental exam was remarkable for tense posture, marked fidgeting, and gaze avoidance. His thought process was illogical regarding emotions and relationships. Affect was remarkable for dysphoria and irritability. His mood was described as “confused.” Insight and judgment were poor.

Hamilton Rating Scale for Depression was 21, severe. A's SCID-2 screen for Borderline Personality was positive with 8/15 items checked. DSM5 criteria were ADHD positive for six inattentive and four hyperactive symptoms. Expired carbon monoxide was 8 part per million reflecting current cigarette use. Cognitive function was perfect. Diagnoses were Major Depressive Disorder, ADHD, Inattentive Type, Tobacco, Cannabis, and Alcohol Use Disorder.

We gave A and his father rating scales. The SRS-2 is validated for adults (Chan et al., 2017). The patient self-rates with a score above 75 indicating severe autism. The SCQ is observer rated; a score of 23.08 is the standardized mean for a diagnosis of autism (Rutter et al., 2003). A scored 79 on the SRS-2. The father rated 27 on the SCQ with a threshold for diagnosis of ASD being 15.

On day 8 cold pressor time (CPT), an ice water bath measuring pain tolerance by timing duration of forearm submersion, was 190 s. Our control average using support persons is 113 s. The withdrawal at 190 s seemed more from annoyance at feeling confined and controlled by remaining stationary rather than intolerable pain. A trial of naltrexone at 25 mg (0.39 mg/kg) was discussed.

A was reluctant to begin naltrexone but provided no reason for his objection. On day 21, A reported improvement in ADHD and depression with twice a week transference focused psychotherapy and bupropion 450 mg/day. After showing these signs of clinical improvement a trial of naltrexone was revisited.

A was apprehensive. He demonstrated an odd rationale about how naltrexone might injure him. We find autistic patients express pride at being autistic, as A did. They speak as if among many frustrations of their life they possess one outstanding quality—being autistic. We clarified his concerns and interpreted rationalizations regarding avoiding treatment. A agreed to begin naltrexone.

On day 28 A started 25 mg naltrexone with improvement in relatedness and odd thinking by day 35. We felt the therapeutic effects of naltrexone 25 mg had plateaued. We increased the dose to 50 mg. A felt significantly better with improved relatedness and clearer thinking.

His CPT was 28 s on 50 mg of naltrexone with a repeat CPT of 39 s a week later. CPT falling from 190 s to 28 or 39 s is thought to represent decreased central nervous system opioid tone, moving A toward the top of the inverse parabolic function in Figure 1. Closeness caused pleasure rather than pain. But A could also feel the pain of emotional responses to relationship problems.

On day 55, A lost his naltrexone prescription. We did not hear about this until inquiring about worsening gaze avoidance, confused thinking, and deterioration of relatedness. At his psychotherapy hour he smelled foul as if self-care had deteriorated.

One evening soon after he cut his left palm with a knife so deeply that the wound required 11 sutures. It was as if A had decided, “I needed to feel pain.” This may have been an attempt to upregulate pain drivers to balance high opioid tone. Again there was reluctance to take medication as his odd thinking prevented appreciation of naltrexone's benefits. His father came to his psychotherapy hour and it was agreed A would restart naltrexone.

On day 65, A returned with vast improvement in mental status on 50 mg of naltrexone. On day 74, A decided to increase naltrexone to 75 mg (1.17 mg/kg). Initially, he felt better. However, after a few days, with no discussion in psychotherapy, he returned to 50 mg. A stated, “At 75 mg I didn't feel like I was on anything.” This is consistent with 75 mg pushing A out of the 2–4 range of Figure 1, and into the 0–1 range of opioid tone where pain is identical to the 5–6 autistic range of opioid tone.

Overall, A felt marked improvement at naltrexone 50 mg. He said, “I can watch a basketball game now with my father without losing interest or wanting drugs or alcohol.” He stopped alcohol, tobacco, and cannabis. Although we attributed the cessation of drug use to his psychotherapy, it may be that naltrexone also made a contribution. Naltrexone is an approved drug for Alcohol Use Disorder. On day 85, A's father rated him a 9 on the SCQ—not consistent with autism. A rated himself 54 on the SRS-2, considered normal.

On day 98, A was hired for a job loading trucks, full time at $14 per hour, and had purchased a new car. He reported positive interactions with coworkers. Soon after he terminated psychotherapy.

On day 166, A had again stopped his naltrexone. He got drunk, crashed his car, and lost his driver's license for a year. A has re-engaged in psychotherapy. Its focus has become negotiating painful relationships including the relational difficulties he encounters with his therapist.

Two years into his psychotherapy A has been working full-time since day 98. He talks mostly about his work relationships. Like other patients with major mental illness, sharing drugs becomes a way of engaging with others. He has resumed inhaling tobacco. He described with great pleasure asking a supervisor for a cigarette. “We walked the whole length of the warehouse to his office. He said, ‘I wouldn't do this for any deadbeat but you work hard so I'll happily give you one of mine.'” Coworkers who inhale tobacco with him on breaks are characterized as, “Good, social people with a lot going on.”

Figures of external authority, such as law enforcement and physicians who, “Always have such a serious look on their face,” are associated with projected judgment and feeling inadequate, evoking guilt and shame. “Court is the worst because everyone is serious. Everyone looks at you like there's something wrong with you.” A's reflective accounts of school are often externalizations he experiences as pondering other people's thoughts of him. He questions whether people past and present were/are honest about their perceptions. This identity question is part of A's transference and a focus of his continuing psychotherapy.

The paranoid thinking about external judgments and malevolence has consistently been examined in the transference, “Are we judging you?” By two years into treatment the paranoia is gone. A is working on developing relationships that he now desires.

## Discussion

Mindful that autism is a heterogeneous disorder with genetic variation, and that there is a range of C-terminal beta endorphin levels in autism, we have presented a neurobiological systems engineering model of “high opioid tone” autism that accounts for many of its features. We have found using the cold pressor test (CPT) to identify autistic persons with presumed high circulating opioids (until measuring C-terminal beta endorphin becomes a routine clinical test) and blocking down pain tolerance using subjective report of feeling better combined with objective lowering of pain tolerance as shown by CPT makes our autistic patients more related and more functional. As shown in this case report, patients who may have gone their whole life with the dysphoria of high opioid tone, face another kind of difficulty when ordinary relatedness, newly experienced, includes the pain induced by having trouble being close to others.

We return to the “three hit model” of autism and describe how our understanding fits the paradigm.

1. The engineering model has nothing to do with genetic predisposition.
2. The second hit, the environmental trigger that interacts with genetic predisposition, is administration of opioids during childbirth. There is no need to administer opioids during childbirth. An epidural block using a non-opioid such as bupivacaine would be adequate. We speculate that the pain of childbirth has a function of setting opioid tone. Administering exogenous hormone disrupts the endogenous opioid system. Producing autism would be another unfortunate, unintended consequence of opioid analgesia.
3. High opioid tone provides an explanation for the diversity of findings in autism.
  - Development is influenced by aberrantly high pain that human interactions provoke. Looking, touching and speaking augment tone which worsens pain, forming the basis of social withdrawal, lack of speech mastery, and gaze avoidance.
  - Parents are in an impossible position. They seek empathic connections ignorant of the reality that empathy relies on everyone having similar brains. Conventional acts of parental love create pain in the child. Any parent would react with confusion when their child recoils from their loving ministrations. Different brains create different responses to human contact.
  - Intestinal problems could be a direct effect of opioids on the gut; analogous with opioid-induced constipation. There is an additional effect of opioids on immune functions located in the gut to prevent bacterial infection that toll-like receptors and opioids modulate. (Rose et al., 2018) High opioid tone may turn on inflammatory cytokines that disrupt gut function.
  - Glial cells in the brain also have toll-like receptors. Endogenous opioids and inflammatory cytokines co-regulate at the toll-like receptors, type 4 (TLR4) (Araldi et al., 2019). High opioid tone may evoke inflammatory cytokines, accounting for brain inflammation in autism (Onore et al., 2012).
  - Endogenous opioids inhibit norepinephrine function in the locus coeruleus that fuel stimulation of corticostriatal pathways (Scavone et al., 2013). Inhibited norepinephrine secretion may result in the high prevalence of ADHD in autism.
  - Disordered sleep is ubiquitous in opioid-maintained persons (Khazie et al., 2016). High opioid tone would account for a similar symptom in autism.
  - Anxiety is a signal that one is distant from loving persons (Watt and Panksepp, 2009). Relationship avoidance to prevent pain creates distance, fitting the high prevalence of anxiety in autism. Autistic persons avoid the pain of closeness at the price of anxiety generated by separation.

If this preliminary model is accepted as having some credence, a new set of investigations is indicated.

1. C-terminal beta endorphin assay is not widely available. Only a few research laboratories measure it. CPT is a stopgap until n-terminal beta endorphin testing becomes commercially available. Naltrexone dose would be determined by amount needed to block down n-terminal beta endorphin into the normal range.
2. Psychotherapy for autism would be tailored to the patient's age/duration of uncorrected high opioid tone.
3. A randomized, double blind evaluation of childbirth with and without opioid analgesia would be undertaken with assessment of gaze-avoidance at an early age, perhaps 12 months. This would confirm or disconfirm the need to discourage opioid administration during childbirth. A broader period of gestation might also be examined, in case opioid tone is set earlier than parturition.
4. Trials of naltrexone with C-terminal beta endorphin assay at different ages during childhood would be undertaken to see if early intervention minimized the damage of high opioid tone to development and relationships.

The authors wish to be clear this is a preliminary hypothesis about the subset of autism high opioid tone may cause. It has the virtue of a parsimonious explanation of a diverse set of attributes of autism.

## Patient Perspective

The patient read the manuscript and gave written permission to use it with some disguising of biographical details but did not care to comment.

## Data Availability Statement

The original contributions presented in the study are included in the article/Supplementary Materials, further inquiries can be directed to the corresponding author/s.

## Ethics Statement

Written informed consent was obtained from the individual(s) for the publication of any potentially identifiable images or data included in this article.

## Author Contributions

VA wrote the first draft. JR wrote the case report. BJ invented the novel concepts in the paper. All authors approved the final work.

## Conflict of Interest

The authors declare that the research was conducted in the absence of any commercial or financial relationships that could be construed as a potential conflict of interest.

## References

[B1] Araldi D. Bogen O. Green P. G. Levine J. D. (2019). Role of nociceptor toll-like receptor 4 (TLR4) in opioid induced hyperalgesia and hyperalgesic priming. J. Neurosci. 14, 6414–6424. 10.1523/JNEUROSCI.0966-19.2019 PMC6697398 31209174

[B2] Campbell M. Anderson L. T. Small A. M. Adams P. Gonzalez N. M. Ernst M. (1993). Naltrexone in autistic children: behavioral symptoms and attentional learning. J. Am. Acad. Child Adolesc. Psychiatry 32, 1283–1291. 10.1097/00004583-199311000-00024 8282676

[B3] Campbell M. Overall J. E. Small A. M. Sokol M. S. Spencer E. K. Adams P.. (1989). Naltrexone in autistic children: an acute open dose range tolerance trial. J. Am. Acad. Child Adolesc. Psychiatry 28, 200–206. 10.1097/00004583-198903000-00009 2925573

[B4] Chan W. Smith L. E. Hong J. Greenberg J. S. Mailick M. R. (2017). Validating the social responsiveness scale for adults with autism. Autism Res. 10, 1663–1671. 10.1002/aur.1813 28639377 PMC5648615

[B5] Grove J. Ripke S. Als T. D.. (2019). Identification of common genetic risk factors for autism spectrum disorder. Nat. Genet. 51, 431–444. 10.1038/s41588-019-0344-8 30804558 PMC6454898

[B6] Hendren R. L. (2020). Integrating treatment for autism spectrum disorders through the life cycle, [Conference Presentation] American Psychoanalytic Association Annual Meeting (New York City, NY). Available online at: https://issuu.com/apsaa/docs/final__-_final_program_2020

[B7] International Council on Systems Engineering. (2006). What is INCOSE's Definition of a SYSTEM? Available online at: https://www.incose.org/docs/default-source/north-star/Ambassador-Program/se-101.pdf?sfvrsn=2andsfvrsn=2 (accessed March 26, 2021).

[B8] Johnson B. (2001). Drug dreams: a neuropsychoanalytic hypothesis. J. Am. Psychoanal. Assoc. 49, 75–96. 10.1177/00030651010490011101 11379730

[B9] Johnson B. (2003). Psychological addiction, physical addiction, addictive character, addictive personality disorder: a new nosology of addiction. Can. J. Psychoanal. 11, 135–160.

[B10] Johnson B. (2008). Just what lies “beyond the pleasure principle”? Neuropsychoanalysis 10, 201–212 10.1080/15294145.2008.10773588

[B11] Johnson B. (2018). Engineering neurobiological systems: addiction. Psychiatr. Clin. North Am. 41, 331–339. 10.1016/j.psc.2018.01.011 29739530

[B12] Johnson B. Flores Mosri D. (2016). The neuropsychoanalytic approach: using neuroscience as the basic science of psychoanalysis. Front. Psychol. 7:1459. 10.3389/fpsyg.2016.01459 27790160 PMC5063004

[B13] Johnson B. Ulberg S. Shivale S. Donaldson J. Milczarski B. Faraone S. V. (2014). Fibromyalgia, autism, and opioid addiction as natural and induced disorders of the endogenous opioid hormonal system. Discov. Med. 18, 209–220. 25336035

[B14] Khazie H. Najafi F. Ghadami M. R. Azami A. Nasouri M. Tahmasian M.. (2016). Sleep disorders in methadone maintenance treatment volunteers and opium-dependent patients. Addict Health 8, 84–89. 27882205 PMC5115641

[B15] Leboyer M. Bouvard M. P. Recasens C. Philippe A. Guilloud-Bataille M.. (1994). Difference between plasma N- and C- terminally directed β-endorphin immunoreactivity in infantile autism. Am. J. Psychiatry 151, 1797–1801. 10.1176/ajp.151.12.1797 7977888

[B16] Lensing P. Schimke H. Klimesch W. Pap V. Szemes G. Klingler D.. (1995). Clinical case report: opiate antagonist and event-related desynchronization in 2 autistic boys. Neuropsychobiology 31,16–23. 10.1159/000119167 7708177

[B17] Onore C. Careaga M. Ashwood P. (2012). The role of immune dysfunction in the pathophysiology of autism. Brain Behav. Immun. 26, 383–392. 10.1016/j.bbi.2011.08.007 21906670 PMC3418145

[B18] Panksepp J. Nelson E. Bekkedal M. (1997). Brain systems for the mediation of social separation-distress and social-reward. Evolutionary antecedents and neuropeptide intermediaries. N. Y. Acad. Sci. 807, 78–100. 10.1111/j.1749-6632.1997.tb51914.x 9071345

[B19] Rose R. Yang H. Serena G. Sturgeon C. Ma B. Careaga H.. (2018). Differential immune responses and microbiota profiles in children with autism spectrum disorders and co-morbid gastrointestinal symptoms. Brain Behav Immunity 70, 354–368. 10.1016/j.bbi.2018.03.025 PMC5953830 29571898

[B20] Rutter M. Bailey A. Lord C. (2003). The Social Communication Questionnaire [Manual]. Torrance, CA: Western Psychological Services.

[B21] Scavone J. L. Sterling R. C. Van Bockstaele E. J. (2013). Cannabinoid and opioid interactions: implications for opiate dependence and withdrawal. J. Neurosci. 248, 637–654. 10.1016/j.neuroscience.2013.04.034 PMC3742578 23624062

[B22] Solms M. Turnbull O. (2002). The Brain and the Inner World: An Introduction to the Neuroscience of Subjective Experience. New York, NY: Other Press.

[B23] Strogatz S. (2019). Infinite Powers: How Calculus Reveals the Secrets of the Universe. New York, NY: Houghton Mifflin Harcourt.

[B24] Watt D. Panksepp J. (2009). Depression: An evolutionarily conserved mechanism to terminate separation distress? A review of aminergic, peptidergic, and neural network perspectives. Neuropsychoanalysis 11, 5–104 10.1080/15294145.2009.10773593

