# Supplementary material for: Autism Case Report: Cause and Treatment of “High Opioid Tone” Autism
Source: Front Psychol. 2021 May 24;12:657952. doi: 10.3389/fpsyg.2021.657952 (PMC8180893; doi:10.3389/fpsyg.2021.657952)
Supplement: Supplementary file 1 [file Table_1.docx]

Supplementary Table

| Year | CDC Prevalence of Autism (per 10,000) | Millions of Opioid Prescriptions (Adjusted*) |
| --- | --- | --- |
| 1991 | 10 | 75 |
| 1995 | 20 | 87 |
| 2001 | 40 | 97 |
| 2004 | 60 | 137 |
| 2007 | 67 | 162 |
| 2010 | 147 | 172 |
| 2014 | 168 | 192 |

Table 1. Population-adjusted rates of autism and opioid prescribing in the United States

1. *Adjustment based on 1991 Population
